# An Exploration of Charge Compensating Ion Channels across the Phagocytic Vacuole of Neutrophils

**DOI:** 10.3389/fphar.2017.00094

**Published:** 2017-02-28

**Authors:** Juliet R. Foote, Philippe Behe, Mathew Frampton, Adam P. Levine, Anthony W. Segal

**Affiliations:** Division of Medicine, Centre for Molecular Medicine, University College LondonLondon, UK

**Keywords:** neutrophil, ion channel, NADPH oxidase, phagocytosis, chloride, potassium

## Abstract

Neutrophils phagocytosing bacteria and fungi exhibit a burst of non-mitochondrial respiration that is required to kill and digest the engulfed microbes. This respiration is accomplished by the movement of electrons across the wall of the phagocytic vacuole by the neutrophil NADPH oxidase, NOX2. In this study, we have attempted to identify the non-proton ion channels or transporters involved in charge compensation by examining the effect of inhibitors on vacuolar pH and cross-sectional area, and on oxygen consumption. The chloride channel inhibitors 4-[(2-Butyl-6,7-dichloro-2-cyclopentyl-2,3-dihydro-1-oxo-1H-inden-5-yl)oxy]butanoic acid (DCPIB) and flufenamic acid (FFA) were the most effective inhibitors of alkalinisation in human neutrophil vacuoles, suggesting an efflux of chloride from the vacuole. The proton channel inhibitor, zinc (Zn^2+^), combined with DCPIB caused more vacuolar swelling than either compound alone, suggesting the conductance of osmotically active cations into the vacuole. Support for cation influx was provided by the broad-spectrum cation transport inhibitors anandamide and quinidine which inhibited vacuolar alkalinisation and swelling when applied with zinc. Oxygen consumption was generally unaffected by these anion or cation inhibitors alone, but when combined with Zn^2+^ it was dramatically reduced, suggesting that multiple channels in combination can compensate the charge. In an attempt to identify specific channels, we tested neutrophils from knock-out mouse models including CLIC1, ClC3, ClC4, ClC7, KCC3, KCNQ1, KCNE3, KCNJ15, TRPC1/3/5/6, TRPA1/TRPV1, TRPM2, and TRPV2, and double knockouts of CLIC1, ClC3, KCC3, TRPM2, and KCNQ1 with HVCN1, and humans with channelopathies involving BEST1, ClC7, CFTR, and MCOLN1. No gross abnormalities in vacuolar pH or area were found in any of these cells suggesting that we had not tested the correct channel, or that there is redundancy in the system. The respiratory burst was suppressed in the KCC3^-/-^ and enhanced in the CLIC1^-/-^ cells, but was normal in all others, including ClC3^-/-^. These results suggest charge compensation by a chloride conductance out of the vacuole and by cation/s into it. The identity of these channels remains to be established.

## Introduction

**FIGURE 1:**
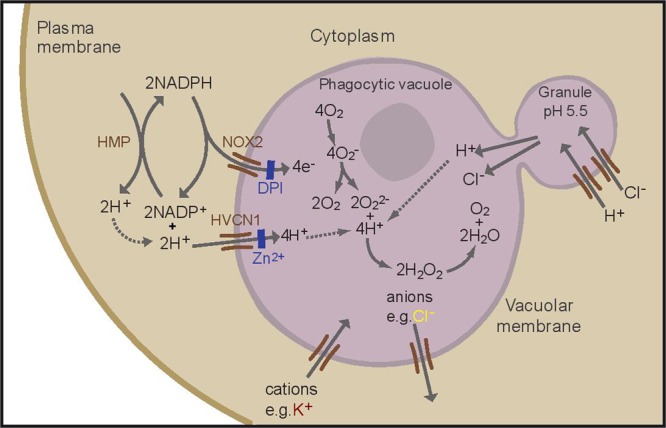
**Schematic representation of the proposed ion fluxes in the neutrophil phagocytic vacuole as a result of oxidase activation.** NOX2 is inhibited by DPI (diphenyleneiodonium), the HVCN1 channel is inhibited by zinc. A proportion of protons present in the cytosol is generated by the hexose monophosphate shunt (HMP). Adapted with permission from (Levine et al., 2015).

The “professional” phagocytes neutrophils, monocytes, eosinophils, and to a lesser extent macrophages, exhibit a profound burst of respiration when they ingest or attach to a microbe (Sbarra and Karnovsky, 1959). This respiratory burst is non-mitochondrial and accomplished by the NADPH oxidase, NOX2, a short electron transport chain that is located in the wall of the phagocytic vacuole, that transfers electrons from NADPH in the cytosol onto oxygen in the vacuole, generating superoxide (Segal and Shatwell, 1997) (**Figure 1**). This electron transfer causes a large and rapid membrane depolarisation which will, itself, curtail further electron transport unless there is compensatory ion movement by the passage of cations into the vacuole and/or anions in the opposite direction (Henderson et al., 1987).

About 90% of this charge compensation is accomplished by the influx of protons (H^+^) through the voltage-gated proton channel HVCN1 (Reeves et al., 2002; Levine et al., 2015). Neutrophils are characterized by an abundance of cytoplasmic granules containing a plethora of enzymes and antimicrobial compounds which are released into the phagocytic vacuole. The cytoplasmic granules have an internal pH of approximately 5, that is maintained by the activity of a V-ATPase (Jankowski et al., 2002), and in the absence of an active oxidase the vacuole becomes acidic when these contents are released into it, as is the case in neutrophils from patients with Chronic Granulomatous Disease (CGD) (Levine et al., 2015), in whom the oxidase is defective. However, it has been recently demonstrated that in normal human neutrophils the vacuolar pH rises to approximately 9.0, a level at which it is maintained for 20–30 min (Levine et al., 2015), which is optimal for the killing and digestion of microbes by neutral proteases, including cathepsin G, and elastase (Levine et al., 2015). This elevation results from the consumption of protons within the vacuole by the protonation of O_2_^2-^ to form H_2_O_2_ (**Figure 1**).

If all the charge were compensated by protons, there would be no nett consumption of these ions, and protons from the fused granules would make the vacuole acidic. Therefore, charge compensation by ions other than protons must occur, and an intriguing aspect of this charge compensation is how the balance of fluxes of the different ions is accomplished to maintain the pH in a fairly precise band of alkalinity. This suggests regulatory gating of proton and non-proton charge compensating ion fluxes based upon pH and charge across the membrane.

To understand this process more fully it is important to identify and characterize the charge compensating channels. There is evidence for the movement of potassium ions (K^+^) into the neutrophil vacuole (Reeves et al., 2002; Rada et al., 2004) and K^+^ channels have been implicated in eosinophils as important for superoxide production (Saito et al., 1995). There is conflicting evidence as to whether Cl^-^ passes into, and/or out of, the phagocytic vacuole. One of the theories concerning the killing of microbes involves the generation of hypochlorous acid (HOCl) by myeloperoxidase from H_2_O_2_ and Cl^-^ (Klebanoff et al., 2013), which would require the passage of Cl^-^ into the vacuole. Some chloride will accumulate in the cytoplasmic granules (Stauber and Jentsch, 2013) to compensate the charge generated when H^+^ are pumped into the granules by a V-ATPase to produce the low pH in these organelles (see **Figure 1**). Macrophages from CLIC1^-/-^ mice display a defect in phagosome acidification (Jiang et al., 2012), and lysosome acidification is defective in alveolar macrophages from mice lacking the cystic fibrosis (CF) transmembrane conductance regulator (CFTR) (Di et al., 2006), a chloride channel, suggesting that these channels might transport Cl^-^ into granules in these cells. CFTR (Painter et al., 2006, 2010), KCC3 (Sun et al., 2012), a K^+^-Cl^-^ co-transporter, and ClC3, a voltage-gated chloride channel that mediates the exchange of chloride ions for protons with a nett movement of Cl^-^ (Moreland et al., 2006), have all been implicated as mechanisms for directly importing Cl^-^ into the vacuole. For CFTR and ClC3 to supply Cl^-^ to the vacuole, it would have to pass against a strong electrochemical gradient whereas KCC3 is electroneutral, so it would not directly act as a charge compensator. Others have suggested that Cl^-^ is passed in the opposite direction, out of the vacuole, through ClC3 (Lassègue, 2007; Miller et al., 2007).

When all electron transport is compensated by protons alone, no change in pH occurs in the vacuole because there is no nett consumption of protons to form H_2_O_2_ (Levine et al., 2015). In addition, and because protons are not osmotically active, there are no consequent changes in vacuolar volume. If, on the other hand, compensation is by ions other than protons, there is an elevation of the vacuolar pH, and changes in the volume will depend upon whether the nett flux of osmotically active ions is into, or out of, the vacuole. Therefore, conclusions as to the nature and direction of the movement of ions can be drawn from changes in pH and vacuolar volume. We recently developed an assay of these parameters, using *Candida albicans* labeled with the fluorescent ratiometric pH indicator seminaphthorhodafluor (SNARF)-1 (Levine et al., 2015). We reasoned that if a single, dominant, non-proton, compensating channel exists, then blocking, or genetically incapacitating, it might alter these parameters.

We tested a range of K^+^ and Cl^-^ channel blockers on normal human neutrophils, and also studied neutrophils from human subjects and mice in which specific ion channels had been deleted by gene targeting, or whose function had been compromised by natural mutation.

In addition to testing those channels previously associated with the oxidase in the literature, we prioritized channels according to mRNA expression levels in human neutrophils. To stress the system and promote ion flux through channels other than the HVCN1 proton channel, we performed studies on neutrophils from knock-out mice lacking both this channel and the channel under investigation, or we conducted the experiment in the presence of zinc chloride, which blocks HVCN1.

## Materials and Methods

### Ethics Approval

This patient study was carried out in accordance with the recommendations of the Joint UCL/UCLH Committees on the Ethics of Human Research with written informed consent from all subjects. All subjects gave written informed consent in accordance with the Declaration of Helsinki. The protocol was approved by the Joint UCL/UCLH Committees on the Ethics of Human Research (Project numbers 02/0324 and 10/H0806/115).

This animal study was carried out in accordance with the recommendations of the United Kingdom Home Office. The protocol was approved by the United Kingdom Home Office (Project licence 70/8452).

### Materials

Balanced salt solution (BSS) buffer contained 156 mM NaCl, 3.0 mM KCl, 1.25mM KH_2_PO_4_, 2 mM MgSO_4_, 2 mM CaCl_2_, 10 mM glucose, 10 mM Hepes at pH 7.4. The KH_2_PO_4_ was removed in experiments employing Zn^2+^ which is precipitated by phosphate.

SNARF was from Invitrogen. Inhibitors were from either Sigma or Tocris and solubilized in dimethyl sulphoxide (DMSO) or 100% ethanol.

### Measurements of Phagosomal pH and Area

These were measured as described previously (Levine et al., 2015). In brief: human neutrophils from peripheral blood were isolated by dextran sedimentation, centrifugation through Lymphoprep, and hypotonic lysis to remove erythrocytes. Neutrophils were resuspended in BSS buffer, and 200 μl of 4 to 5 × 10^6^ cells/ml were incubated for 45 min in the wells of a poly L-lysine coated Ibidi μ-Slide 8 well plate (Ibidi, Germany) to form an adherent cell monolayer.

The wells were washed twice with BSS buffer, and 200 μl buffer containing 1 μg carboxy SNARF-1, AM ester, acetate in 1 μl DMSO was added for 25 min to label the cytosol. After washing the wells twice, the incubation medium consisting of BSS buffer was added with or without the inhibitor.

Heat-killed *Candida* labeled with SNARF and opsonized with human serum IgG (Vivaglobin), were added to the wells. The plates were incubated at 37°C for 20 min, after which confocal images were obtained with a Zeiss 700 confocal microscope using a 63× oil immersion with excitation at 555 nm and emission at 560–600 nm and >610 nm.

For neutrophils isolated from murine bone marrow, cells were flushed from the bone marrow of the tibias and femora with BSS buffer containing 0.5 mM EDTA, 5 IU/ml Heparin and 10% bovine serum albumin, and purified as described above. The *Candida* particles used for phagocytosis were opsonized with a mixture of normal mouse serum (VWR) and serum from mice immunized with *Candida* as described previously (Levine et al., 2015).

The vacuolar pH was measured using a custom macro within the imaging software ImageJ (Schneider et al., 2012). The SNARF fluorescence ratio (SFR) values were converted to pH using the standard curves as described by Levine et al. (2015): the fluorescence ratios of extracellular SNARF-labeled *Candida* were measured in different buffer solutions (100 mM KCl with 50 mM buffer salt) from pH 3–13 to construct two standard curves; the fluorescence ratios of SNARF-labeled *Candida* engulfed by human neutrophils were measured after the phagocytosing cells were then subjected to the same buffers with 0.3% saponin; cytoplasmic pH was measured in human neutrophils in the same buffers with nigericin (Morgan et al., 2009). The outer perimeter of vacuoles containing one visible yeast particle was manually delineated. The cross-sectional area was then determined with ImageJ.

### Analysis of Phagosomal pH and Area

We found that human vacuolar pH measurements were distributed into two main groups, alkaline, and more acidic. This can be explained because vacuolar closure is incomplete in some cases, and because the timing of particle uptake was not synchronized, so some would have been older than others. In addition, as seen in Figure 3E of Levine et al. (2015) representing a time course of human neutrophil phagocytosis, some vacuoles returned to more acidic levels within 30 min, after initially becoming alkaline.

Due to our interest in the factors influencing the alkalinisation of the vacuole, we undertook a cluster analysis of the control vacuolar data, using the package ‘Mclust’ (Fraley et al., 2012) in the programming software R (R Core Team, 2013). This fits data into Gaussian finite mixture models with an algorithm for model-based clustering, classification, and density estimation. The maximum measurement of the lower ratio acidic group was calculated, and points below this value were removed (see figure legends for exact number). HVCN1^-/-^ murine neutrophils also have a smaller subset of acidic vacuoles which were removed by the same method. This particular analysis was not necessary for wild type mouse neutrophil vacuolar pH as they tended to be more acidic and not readily separated into two clusters.

For both vacuolar pH and area, the measurements have been displayed as scatter plots with overlaying boxplots, which show the median and upper and lower quartiles. Linear model analysis, using the lm function in the programming software R (R Core Team, 2013), was used to test the statistical significance of the comparison between the control condition and that in the presence of the inhibitor, or in cells with an altered genetic background.

### Measurement of Respiratory Burst

Respiration was measured as described previously (Levine et al., 2015) with the Seahorse Bioscience XFe24 Extracellular Flux Analyzer. A calibration plate was first run through the machine according to the manufacturer’s instructions. The assay plate was pre-treated with 50 μl of 22.4 μg/ml Cell-Tak (BD Bioscience) in PBS in each well for 45 min at room temperature and washed twice with sterile water. Hundred microliter of a suspension of 4 × 10^7^/ml human neutrophils, resuspended in the BSS buffer lacking phosphate, was added to each well. The plate was centrifuged first at 450 g for 1 min and then for a minute in the opposite orientation. The medium was replaced with 600 μl of the BSS buffer lacking phosphate with or without the inhibitor. 4 × 10^6^ in 10 μl of opsonized *Candida* was added to each well then readings commenced. The inhibitor was present in the medium before and after the addition of *Candida* so it could access both cytoplasmic and vacuolar membranes. The oxygen consumption was calculated as the integral of the rate of oxygen consumption for 15 min

Measurement of oxygen consumption was conducted on mouse neutrophils isolated from the peritoneal cavity after stimulation by thioglycolate (as described previously; Levine et al., 2015), as described above using 4 × 10^6^ cells/ml.

Statistical analysis was calculated in Excel by Student’s *t*-test of paired means across the individual wells within each experiment and across repeats of the same experiment.

### Gene Expression Profiling of Human Neutrophils

Gene expression data from resting human neutrophils from healthy individuals were isolated from nine individual publically available studies on the Gene Expression Omnibus (Edgar et al., 2002) (Supplementary Table S2). There were data from a total of 66 samples, a mean of seven (minimum three) per study. Data were analyzed with R (R Core Team, 2013). For all but one dataset, raw data were processed using the Affy package (Gautier et al., 2004). For one dataset, pre-processed data were used. Raw data were imported using ReadAffy and normalized with Robust Multi-array Average. Detected probes were then identified per sample using pa.calls from the panp package (Warren, 2016). Probes that were detected in at least 50% of the individual samples within each data set were retained. Genes within a gene family (Gray et al., 2015) containing the terms channel or carrier or those with a GO term containing one of the following were retained: potassium, sodium, chloride, channel, or exchanger. Control genes that were known to be involved in neutrophil function or markers of possible contaminating cells were also retained. Within each dataset, the relative centile expression of the retained genes from all detected genes was determined. The summary statistics across the nine studies were combined by gene and the mean calculated. It is conceivable that a putative channel or exchanger involved in neutrophil charge compensation could have been inappropriately omitted by the requirement for either the gene family or GO term to be matched; however, this approach was utilized as a means of reducing the search space to contain a manageable number of the most likely candidates.

### Sources of Neutrophils

#### Mutant Mice

We studied knockout mice lacking CLIC1 (Qiu et al., 2010), KCC3 (Lucas et al., 2012), ClC3 (Stobrawa et al., 2001), KCNQ1 (Frohlich et al., 2011), KCNE3 (Roepke et al., 2011), KCNJ15 (MRC Harwell), TRPM2 (Yamamoto et al., 2008), TRPV2 (Link et al., 2010), a mouse lacking the combination of TRPC1, TRPC3, TRPC5 and TRPC6 and a dual KO of TRPV1 and TRPA1, SLC26A6. We also studied a mouse with a loss of function mutation in ClC7 (Alam et al., 2014). We bred the HVCN1/ CLIC1, HVCN1/ ClC3, HVCN1/KCC3, HVCN1/ TRPM2, HVCN1/ KCNQ1, and TRPM2/KCNQ1 double knock-outs. The sources of all mice are provided in the Acknowledgments section.

#### Patients with Channelopathies

We studied human cells from individuals with mutations in CFTR (two patients both homozygous ΔF508), BEST1 (two heterozygote patients with Best vitelliform macular dystrophy due to mutations in BEST1: (patient 1) a 25 year old female heterozygous for a missense (p.H178P) and frameshift (p.Tyr347LeufsX54) mutations, and (patient 2) a 55 year-old male with total allelic deletion p.Pro152Ala,p.Ala195Val). For patients with a mutation in ClC7 (Albers-Schönberg disease; patient 1 with homozygous novel missense change c.1120G > A, p.(Glu374Lys) in exon 13; patient 2 with a homozygous novel missense change at c.2332-IG > A in intron 24 which lead to defective splicing of exon 24 and 25). One patient with a mutation in MCOLN1 (Mucolipidosis type IV c.236_237ins93 from NADH dehydrogenase 5 99-192 and R403C) was recruited.

## Results

### The Effect of Ion Channel Inhibitors on Human Vacuolar pH and Swelling

#### Chloride Channel Inhibitors Cause Significant Vacuolar Acidification

**FIGURE 2:**
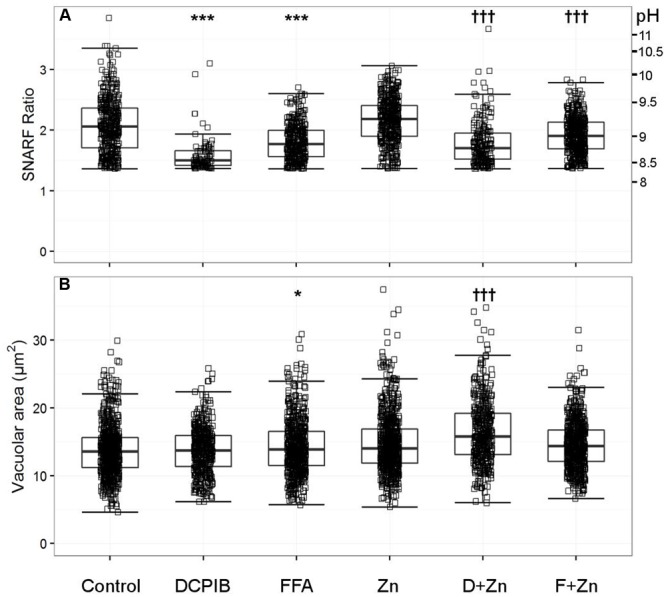
**The effect of DCPIB and FFA with and without zinc on human vacuolar pH (A)** and area **(B)**. The acidic vacuole population was removed as described in the methods section; the vacuoles excluded are those with a SNARF Fluorescence ratio (SFR) below 1.36. Each point represents an individual measurement (see **Table 1** for the exact number of measurements and statistics); each condition has an overlaying boxplot showing the median and interquartile range. DCPIB (20 μM), FFA (100 μM), Zn (Zn^2+^ 100 μM), D+Zn (DCPIB with Zn^2+^), F+Zn (FFA with Zn^2+^). Differences from control neutrophils: ^∗^*p* < 0.05, ^∗∗^*p* < 0.01, ^∗∗∗^*p* < 0.001. Differences from neutrophils incubated with Zn2+: †*p* < 0.05, ††*p* < 0.01, †††*p* < 0.001.

In human cells, the broad spectrum chloride channel blockers DCPIB and FFA caused the most significant abrogation of vacuolar alkalinisation. Of the many classical ion channel inhibitors tested (see Supplementary Table S1), DCPIB was the most effective in this manner by decreasing the pH from ∼ 9.0 to 8.5 (**Figure 2A**). FFA also caused a smaller, but still statistically significant, decrease to ∼ pH 8.8. Zn^2+^ used at 100 μM increase the vacuolar pH to ∼ pH 9.2. The elevation in pH and vacuolar area induced by Zn^2+^ was still much less than that resulting from complete elimination of the channel in the HVCN1^-/-^ cells, indicating that Zn^2+^ only caused a partial blockage.

In the presence of 100 μM Zn^2+^, DCPIB and FFA also reduced the vacuolar pH to lower than the Zn^2+^ control; ∼ pH 8.8 and 9.0 respectively (**Figure 2A**). These findings suggest that a Cl^-^ channel is likely to be involved in charge compensation.

#### Chloride Channel Inhibitors Increase Vacuolar Swelling

DCPIB alone had no effect on the vacuolar area (**Figure 2B**), whereas FFA increased this slightly from 13.4 to 13.9 μm^2^. However, the *p*-value of 0.016 was relatively insignificant when adjusting for multiple testing – we investigated nearly 70 different ion channel inhibitors which naturally lowers the significance threshold when using the Bonferroni correction. Zn^2+^ alone increased swelling to 13.9 μm^2^ but had a more statistically significant *p*-value of 1.25 × 10^-8^; see **Table 1B** for more detailed statistics. When Zn^2+^ and FFA were combined the median vacuolar size was not significantly different from Zn^2+^ alone, however, when DCPIB was combined with Zn^2+^ vacuolar size increased dramatically to 15.8 μm^2^. The increased swelling of vacuoles induced by chloride channel blockers suggests that chloride is being retained within the vacuole and/or its export replaced, at least in part, by the influx of an osmotically active cation.

**Table 1:** Vacuolar pH **(A)** and area **(B)** experimental measurements and statistics for chloride channel inhibitors.

(A) Vacuolar SNARF ratio					
***N***	**Control**	**No. of points**	**Control median (SFR/pH)**		**Inhibitor**	**No. of points**	**Inhibitor median (SFR/pH)**		***p*-value**

11	Control	875	2.0	9.0	Zinc	1014	2.2	9.2	7.75 × 10^-13^
3	Control	202	1.9	9.0	DCPIB	85	1.5	8.5	3.53 × 10^-12^
4	Control	330	2.1	9.1	FFA	333	1.8	8.8	3.73 × 10^-24^
3	Zinc	227	2.2	9.2	DCPIB + zinc	187	1.7	8.8	1.17 × 10^-14^
4	Zinc	353	2.2	9.2	FFA + zinc	443	1.9	9.0	2.32 × 10^-25^

**(B) Vacuolar area**					

***N***	**Control**	**No. of points**	**Control median (±*SD*)**		**Inhibitor**	**No. of points**	**Inhibitor median (±*SD*)**		***p*-value**

11	Control	1537	13.2 (3.8)		Zinc	1574	13.9 (4.4)		1.25 × 10^-8^
3	Control	454	13.4 (3.7)		DCPIB	429	13.7 (3.4)		0.145
4	Control	523	13.4 (3.8)		FFA	576	13.9 (4.1)		0.016
3	Zinc	380	13.6 (4.8)		DCPIB + zinc	406	15.8 (4.7)		2.52 × 10^-7^
4	Zinc	560	13.8 (3.9)		FFA + zinc	581	14.3 (3.6)		0.093

*N = number of experiments performed, no. of points describes the total number of individual measurements. The control and corresponding knockout medians are accompanied with the SFR and converted vacuolar pH and standard deviation (SD) for the vacuolar area.*

#### Potassium Channel Inhibitors Cause a Small Reduction in Vacuolar pH

**FIGURE 3:**
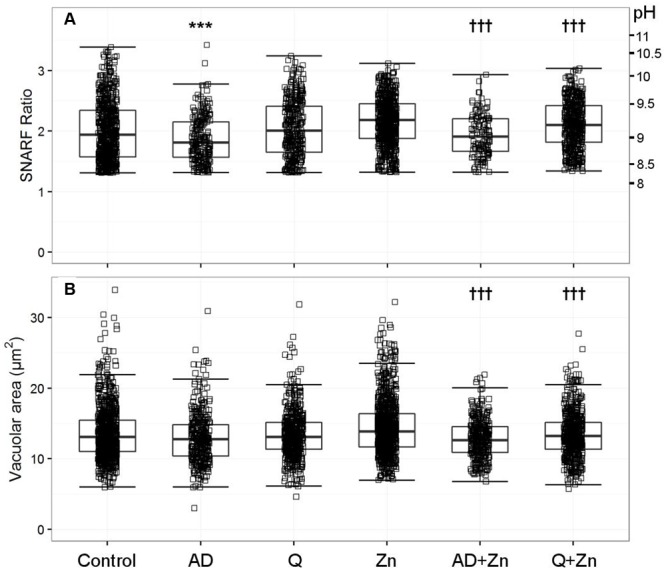
**The effect of anandamide and quinidine with and without Zn^2+^ on vacuolar pH (A)** and area **(B).** The acidic vacuoles excluded are those with a SFR below 1.31. Each square represents an individual measurement; each condition has an overlaying boxplot showing the median and interquartile range. More detailed statistics are provided in **Table 2**. A (anandamide 100 μM), Q (quinidine 100 μM), Zn (Zn^2+^ 100 μM). Differences from control neutrophils: ^∗^*p* < 0.05, ^∗∗^*p* < 0.01, ^∗∗∗^*p* < 0.001. Differences from neutrophils incubated with Zn^2+^: †*p* < 0.05, ††*p* < 0.01, †††*p* < 0.001.

Anandamide is a non-specific blocker of voltage-gated and two-pore K^+^ channels. It caused a slight vacuolar acidification alone (**Figure 3A**), from ∼ pH 9.1 to 8.9, and also when combined with Zn^2+^, from ∼ pH 9.2 to 9.0. Quinidine only caused a small drop in vacuolar pH when combined with Zn^2+^ (∼ pH 9.2 to 9.1). It is also a broad-spectrum inhibitor of several potassium channels, sodium channels, and the swell-activated chloride channel (ICI_swell_). However, the effect of both these inhibitors on vacuolar pH was much smaller than with DCPIB or FFA, **Table 2** describes the complete measurements and statistics. **Table 3** lists the purported sites of inhibition and activation of the other compounds tested and their general effects on the vacuolar parameters.

**Table 2:** Vacuolar pH **(A)** and area **(B)** experimental measurements and statistics for potassium channel inhibitors.

(A) Vacuolar SNARF ratio					
***N***	**Control**	**No. of points**	**Control median (SFR/pH)**		**Inhibitor**	**No. of points**	**Inhibitor median (SFR/pH)**		***p*-value**

3	Control	271	2.1	9.1	Anandamide	230	1.8	8.9	1.64 × 10^-5^
4	Control	342	1.9	8.9	Quinidine	348	2.0	9.1	0.264
3	Zinc	308	2.2	9.2	Anandamide + zinc	128	1.9	9.0	1.12 × 10^-6^
4	Zinc	372	2.2	9.2	Quinidine + zinc	461	2.1	9.1	3.88 × 10^-6^

**(B) Vacuolar area**					

***N***	**Control**	**No. of points**	**Control median (±*SD*)**		**Inhibitor**	**No. of points**	**Inhibitor median (±*SD*)**		***p*-value**

3	Control	336	12.8 (3.5)		Anandamide	346	12.8(3.6)		0.085
4	Control	374	13.2 (3.9)		Quinidine	484	13.1(3.4)		0.364
3	Zinc	392	14.2 (4.0)		Anandamide + zinc	324	12.6 (2.7)		6.11 × 10^-14^
4	Zinc	470	13.9 (4.0)		Quinidine + zinc	522	13.2(3.2)		2.54 × 10^-4^

*N = number of experiments performed, no. of points describes the total number of individual measurements. The control and corresponding knockout medians are accompanied with the SFR and converted vacuolar pH and standard deviation (SD) for the vacuolar area.*

**Table 3:** Summary of the effects of specified compounds with their reported sites of activity, and the general effect on vacuolar pH and area in human neutrophils.

Compound	Sites of inhibition (specific channel/transporter or conductance)	Sites of activation	Effect on neutrophils compared to control	
				+ zinc 100 μM
DCPIB	ICI_swell_ (Decher et al., 2001), Kir (Deng et al., 2016), KCC3 (Adragna et al., 2015)	TREK-1,-2 (Minieri et al., 2013)	↓ pH	↓ pH ↑ area
FFA	ICI_swell_ (Jin et al., 2003), Ca^2+^ activated Cl^-^ (Liantonio et al., 2007; Oh et al., 2008), TRP (Guinamard et al., 2013)	TREK-1,-2 (Takahira et al., 2005)	↓ pH ↑ area	↓ pH
Anandamide	TASK-1, -3 (2P K channels) (Maingret et al., 2001), Kv channels (Poling et al., 1996)	TRPV1 (Jerman et al., 2002)	↓ pH	↓ pH ↓ area
Quinidine	Na^+^ channels (Hondeghem and Matsubara, 1988), KCa channels (Iwatsuki and Petersen, 1985) Kv channels (Yatani et al., 1993) 2P K channels (Patel et al., 2000) ICI_swell_ (Voets et al., 1996)			↓ pH ↓ area

#### Potassium Channel Inhibitors Reduce Vacuolar Swelling

In contrast to the effect of the chloride channel blockers, anandamide and quinidine caused vacuolar shrinking - but only when applied with Zn^2+^ (**Figure 3B**). Anandamide with Zn^2+^ was the most effective condition, causing a reduction in vacuolar area from 13.9 to 12.8 μm^2^, while the median vacuolar area of quinidine with Zn^2+^ was 13.2 μm^2^. This suggests that an osmotically active ion is being prevented from entering the vacuole.

### Testing the Effect of Ion Channel Inhibitors on the Respiratory Burst of Human Neutrophils

#### Chloride Channel Inhibitors Decrease the Respiratory Burst Only When Applied with Zn^2+^

**FIGURE 4:**
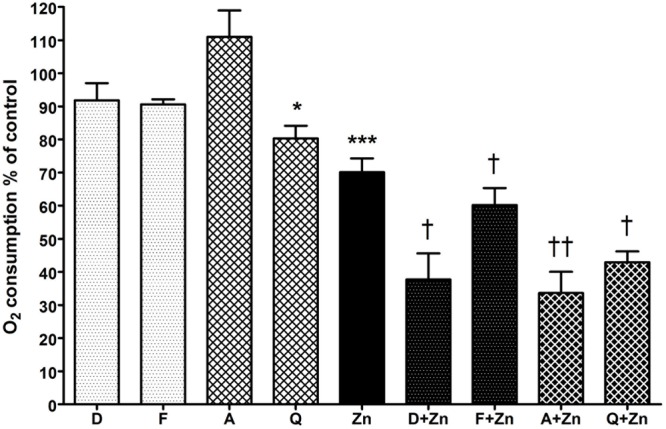
**The effect of DCPIB, FFA, anandamide, and quinidine with and without Zn^2+^ on human neutrophil oxygen consumption during phagocytosis of opsonized *Candida* particles.** The columns represent the mean of 3 experiments as a percentage of neutrophils incubated with the vehicle only, with error bars showing the standard error. Differences from control neutrophils: ^∗^*p* < 0.05, ^∗∗∗^*p* < 0.001. Differences from neutrophils incubated with Zn^2+^: †*p* < 0.05, ††*p* < 0.01. D (DCPIB 20 μM), F (FFA 100 μM), A (anandamide 100 μM), Q (quinidine 100 μM), Zn (Zn^2+^ 100 μM).

To determine if the drop in vacuolar pH caused by DCPIB and FFA was due to direct inhibition of the NADPH oxidase, the respiratory burst was measured by recording oxygen consumed after phagocytosis of opsonized heat-killed *Candida* particles (**Figure 4**). Neither drug had a statistically significant effect on oxidase activity, although the percentage mean oxygen consumed was slightly lower than control cells: DCPIB 91.8% (SEM ± 5.2%), while FFA marginally decreased it further (90.6 ± 1.5%). However, the respiratory burst activity was considerably diminished when the drugs were incubated with Zn^2+^: DCPIB plus Zn^2+^ induced a drop to 37.7% (± 7.9%, *p* = 0.01), while the effect was less pronounced with FFA with Zn^2+^ (60.2 ± 5.1%, *p* = 0.038). These results suggest that blockage of HVCN1 and Cl^-^ channel/s together markedly impaired charge compensation.

#### Potassium Channel Inhibitors Only Significantly Decrease the Respiratory Burst When Applied with Zn^2+^

Anandamide and quinidine had different effects on vacuolar pH and area from the chloride channel inhibitors, but the effect on respiratory burst activity was fairly similar (**Figure 4**). With anandamide, oxygen consumed increased slightly to 111% (±8.0%, *p* = ns) whereas quinidine produced a small but significant effect with a drop of mean O_2_ consumption to 80.3% (±3.8%, *p* = 0.02). Zn^2+^ alone reduced respiration to about 70% of control as previously reported (DeCoursey et al., 2003) whereas in combination with anandamide or quinidine it further decreased respiration, falling to 33.6% (±6.4%, *p* = 0.005) and 42.9% (±3.3%, *p* = 0.01) respectively.

### Vacuolar pH and Areas of Neutrophils from Genetically Modified Mice

#### Single and Double Knockout Mice Tested have Grossly Normal Vacuolar pH and Area

**FIGURE 5:**
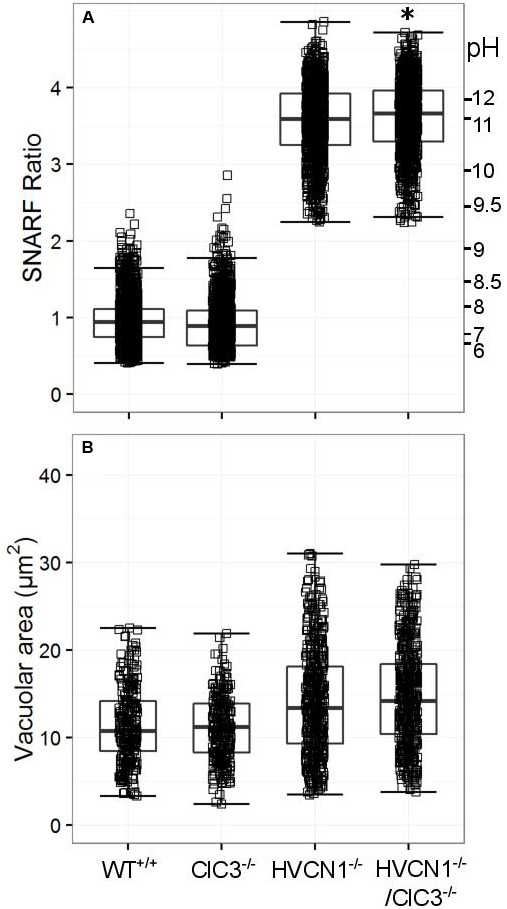
**Vacuolar pH (A)** and area **(B)** measured in the single knockout ClC3^-/-^ mice, and in the double knockout HVCN1^-/-^/ClC3^-/-^ mice. Each square represents an individual measurement with an overlaying boxplot showing the median and interquartile range. The SFR cut-off value for the HVCN1^-/-^ and double knockout mice was 2.23. See **Table 5** for more detailed statistics. Differences from control neutrophils: ^∗^*p* < 0.05.

ClC3 was found to be significantly expressed in the neutrophil mRNA composite data (**Table 4**), and regarded by some (as discussed in the introduction) to be involved in neutrophil oxidase charge compensation (Moreland et al., 2006). We found no abnormalities in the vacuolar pH for ClC3^-/-^, but HVCN1^-/-^/ClC3^-/-^ neutrophils had a slightly higher vacuolar pH (**Figure 5A**). Additionally, the difference between the HVCN1^-/-^ control and double knockout was only 0.1 pH unit (see **Table 5A** for exact statistics). The vacuolar area was not significantly different between either condition (**Figure 5B**).

**Table 4:** A compilation of the averaged results of nine online datasets of mRNA expression in neutrophils.

Gene symbol	Gene description	Relative expression (Human)	Human	Mouse
**Oxidase components**				
NCF2	Neutrophil cytosolic factor 2	98.50		
CYBA	Cytochrome b-245, alpha polypeptide	86.79		
NCF4	Neutrophil cytosolic factor 4, 40 kDa	94.99		
CYBB	Cytochrome b-245, beta polypeptide	61.05		
HVCN1	Hydrogen voltage-gated channel 1	78.57		
**Chloride channels**				
CLIC1	Chloride intracellular channel 1	95.92	Averaimo et al., 2010	^∗^
BEST1	Bestrophin 1	59.91	^∗^Fischmeister and Hartzell, 2005	
CIC7	Chloride channel, voltage-sensitive 7	41.53	^∗^Graves et al., 2008	^∗^
ClC3	Chloride channel, voltage-sensitive 3	35.36	Moreland et al., 2006	^∗^Moreland et al., 2006
**Potassium channels**				
KCNJ15	Potassium inwardly-rectifying channel, subfamily J, member 15	93.69		^∗^
KCNE3	Potassium voltage-gated channel, Isk-related family, member 3	77.44	Barro-Soria et al., 2015	^∗^
KCNJ2	Potassium inwardly-rectifying channel, subfamily J, member	80.66	^∗^Vicente et al., 2003	Masia et al., 2015
KCNQ1	Potassium voltage-gated channel, KQT-like subfamily, member 1	48.37		^∗^
KCNAB2	Potassium voltage-gated channel, shaker-related subfamily, beta member 2	43.76		
KCNK7	Potassium channel, subfamily K, member 7	41.97		
KCNH7	Potassium voltage-gated channel, subfamily H (eag-related), member 7	32.57		
KCNH3	Potassium voltage-gated channel, subfamily H (eag-related), member 3	7.02		
**Transient receptor potential channels**				
TRPM6	Transient receptor potential cation channel, subfamily M, member 6	31.06		
MCOLN1	Mucolipin 1	31.60	^∗^Bach et al., 1999	
TRPV2	Transient receptor potential cation channel, subfamily V, member 2	17.94		^∗^Link et al., 2010
TRPV6	Transient receptor potential cation channel, subfamily V, member 6	11.15	Heiner et al., 2003b	
**Solute carriers**				
SLC12A6 (KCC3)	Solute carrier family 12 (potassium/chloride transporters), member 6	92.47	Sun et al., 2012	^∗^Sun et al., 2012
SLC26A6	Solute carrier family 26, member 6	29.45		^∗^Ishiguro et al., 2006
SLC12A4 (KCC1)	Solute carrier family 12 (potassium/chloride transporters), member 4	14.34		

*Genes were ranked on relative mean expression levels; a more detailed explanation for acquiring the data is described in the methods. Expression levels of components of the neutrophil NOX2 oxidase system, gp91phox, p22phox, NCF1, NCF2, and HVCN1 are shown for comparison. The table has been edited to show channels linked to neutrophils, NOX systems or other immune cells in the literature. Asterisks denote which genetic models we have tested. Shaded rows indicate genetic models not yet investigated. The source of each data set can be found in Supplementary Table S5, while the full list of gene expression can be viewed in Supplementary Table S6. We also tested CFTR, ClC4, TRPA1, TRPC1, TRPC3, TRPC5, TRPC6, TRPM2, and TRPV1 for which expression levels were very low or non-existent.*

**Table 5:** Vacuolar pH **(A)** and area **(B)** experimental measurements and statistics for the knockout mouse models presented in **Figures 5**–**8**.

(A) Vacuolar SNARF ratio					
**N**	**Control**	**No. of points**	**Control median (SFR/pH)**		**Knockout**	**No. of points**	**Knockout median (SFR/pH)**		***p*-value**

3	Wt	1269	0.9	7.5	ClC3^-/-^	1116	0.9	7.5	0.268
2	HVCN1^-/-^	1130	3.6	11.1	HVCN1^-/-^/ClC3^-/-^	1157	3.7	11.2	0.020
4	Wt	1637	0.9	7.4	KCC3^-/-^	1340	0.9	7.5	2.0 × 10^-4^
3	HVCN1^-/-^	383	2.8	9.8	HVCN1^-/-^/KCC3^-/-^	472	2.8	9.9	0.165
3	Wt	589	1.2	8.0	CLIC1^-/-^	780	1.2	8.0	0.598
3	HVCN1^-/-^	1594	3.5	10.8	HVCN1^-/-^/CLIC1^-/-^	1596	3.5	10.8	0.174
3	Wt	371	1.0	7.8	TRPM2^-/-^	399	1.0	7.8	0.087
1	HVCN1^-/-^	118	1.9	9.0	HVCN1^-/-^/TRPM2^-/-^	82	1.8	8.9	0.120
3	Wt	898	1.0	7.8	KCNJ15^-/-^	727	1.1	7.9	0.016

**(B) Vacuolar area**					

**N**	**Control**	**No. of points**	**Control median (±*SD*)**		**Knockout**	**No. of points**	**Knockout median (±*SD*)**		***p*-value**

3	Wt	281	10.8 (4.9)		ClC3^-/-^	273	11.2 (4.5)		0.650
2	HVCN1^-/-^	549	13.4 (6.7)		HVCN1^-/-^/ClC3^-/-^	570	14.2 (6.1)		0.109
3	Wt	445	11.2 (3.4)		KCC3^-/-^	458	11.3 (3.9)		0.469
3	HVCN1^-/-^	389	16.7 (6.4)		HVCN1^-/-^/KCC3^-/-^	287	17.3 (5.5)		0.554
3	Wt	266	12.9 (4.2)		CLIC1^-/-^	346	13.2 (4.5)		0.167
3	HVCN1^-/-^	696	15.1 (7.4)		HVCN1^-/-^/CLIC1^-/-^	650	12.8 (6.8)		6.7x10^-7^
3	Wt	162	11.7 (5.3)		TRPM2^-/-^	208	11.0 (5.1)		0.569
1	HVCN1^-/-^	78	17.1 (9.9)		HVCN1^-/-^/TRPM2^-/-^	39	18.7 (11.4)		0.830
3	Wt	633	9.9 (3.9)		KCNJ15^-/-^	387	10.4 (4.6)		0.003

*N = number of experiments performed, no. of points describes the total number of individual measurements. The control and corresponding knockout medians are accompanied with the SFR and converted vacuolar pH and standard deviation (SD) for the vacuolar area.*

**FIGURE 6:**
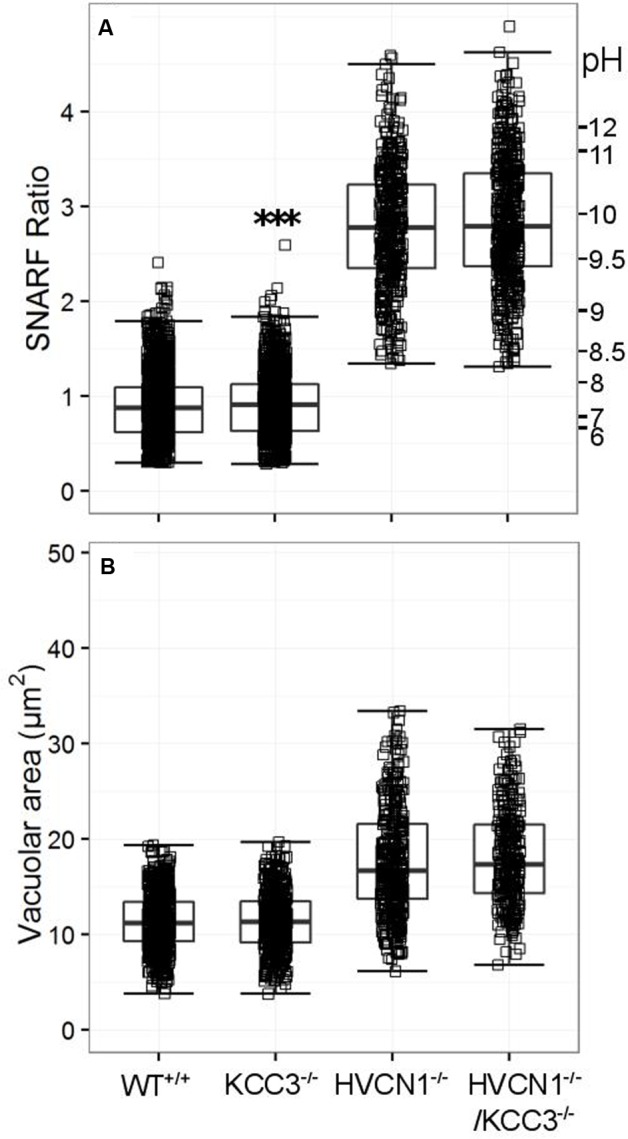
**Vacuolar pH (A)** and area **(B)** measured in the single knockout KCC3^-/-^ mice, and in the double knockout HVCN1^-/-^/KCC3^-/-^ mice. Each square represents an individual measurement with an overlaying boxplot showing the median and interquartile range. A small difference was found between the vacuolar pH of WT and KCC3^-/-^ neutrophils which was statistically significant (^∗∗∗^*p* < 0.001). The SFR cut-off value for the HVCN1^-/-^ and double knockout mice was 1.31.

No significant change in vacuolar area was found for either KCC3^-/-^ or HVCN1^-/-^/KCC3^-/-^ (**Figure 6A**), but the vacuolar pH was slightly increased in the single knockouts (**Figure 6B**), from ∼ pH 7.4 to 7.5.

**FIGURE 7:**
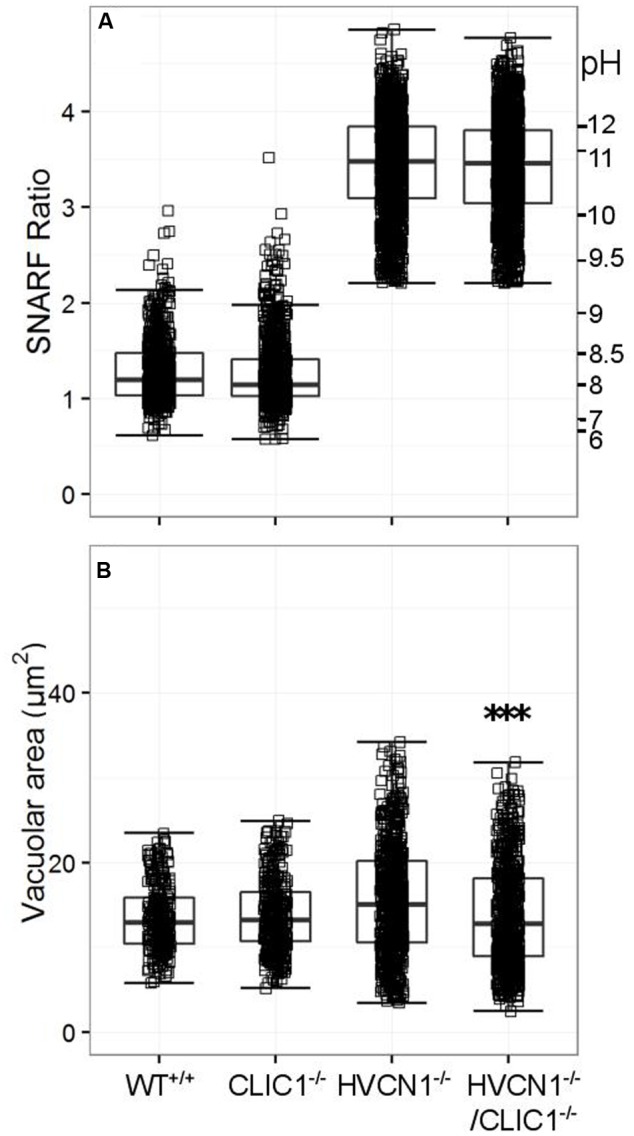
**Vacuolar pH (A)** and area **(B)** measured in the single knockout CLIC1^-/-^ mice, and in the double knockout HVCN1^-/-^/CLIC1^-/-^ mice. Each square represents an individual measurement with an overlaying boxplot showing the median and interquartile range. The SFR cut-off value for the HVCN1^-/-^ and double knockout mice was 2.20. Difference between HVCN1^-/-^ and HVCN1^-/-^/CLIC1^-/-^: ^∗∗∗^*p* < 0.001.

While CLIC1 has not been directly linked to neutrophil oxidase activity, it is thought to be involved with lysosomal acidification in macrophages (Jiang et al., 2012), and it was highly expressed in the composite expression data (**Table 4**). We found no change in vacuolar pH in either model compared to the wild type (WT) or HVCN1^-/-^ controls (**Figure 7A**), but there was a highly significant decrease in vacuolar area in the HVCN1^-/-^/CLIC1^-/-^ neutrophils; the median decreased from 15.1 μm^2^ to 12.8 μm^2^.

**FIGURE 8:**
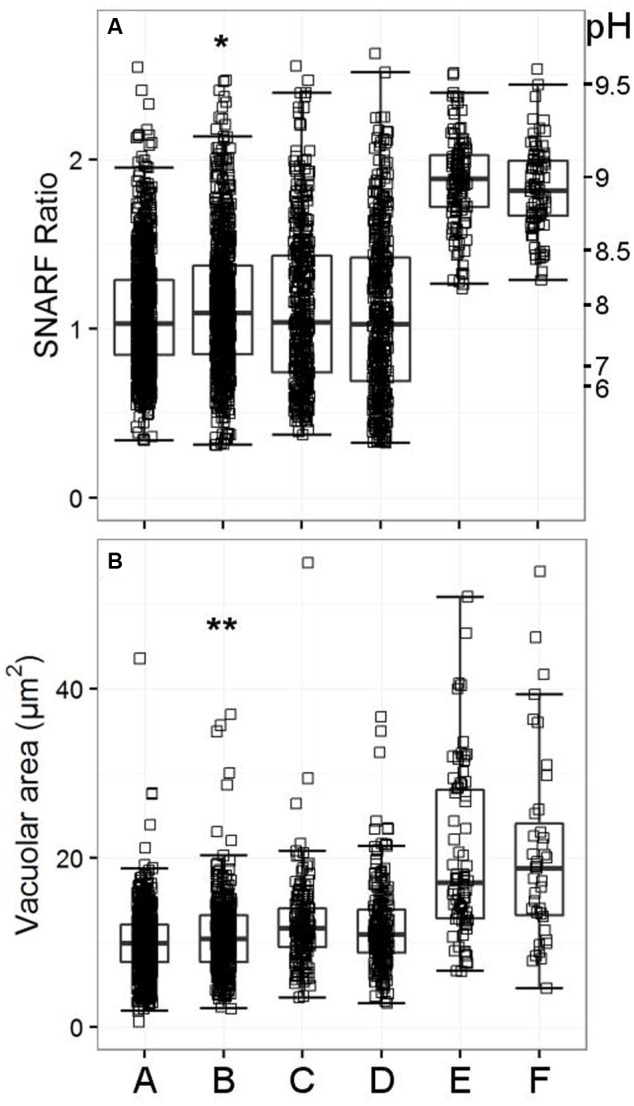
**Vacuolar pH (A)** and area **(B)** measured in the single knockout KCNJ15^-/-^ mice, single knockout TRPM2^-/-^ mice, and in the double knockout HVCN1^-/-^/TRPM2^-/-^ mice. The SFR cut-off value for the HVCN1^-/-^ and double knockout mice was 1.197. A (WT used for KCNJ15^-/-^), B (KCNJ15^-/-^), C (WT used for TRPM2^-/-^), D (TRPM2^-/-^), E (HVCN1^-/-^), F (HVCN1^-/-^/TRPM2^-/-^). Differences between WT and KCNJ15^-/-^ neutrophils: ^∗^*p* < 0.05, ^∗∗^*p* < 0.01.

TRPM2 has been cited as being important in negative feedback of the NADPH oxidase, yet we could find no abnormalities in either vacuolar pH or area for the single knockout or double knockout (HVCN1^-/-^/TRPM2^-/-^) mice (see **Figure 8**).

Because the inhibitor studies indicated that Cl^-^ and cation channels were likely to be involved in charge compensation we decided to test those most highly expressed in neutrophils as shown in **Table 4**. We did not find anything that was significantly abnormal in these other knockout mouse models (although some were only tested once, further details in Supplementary Tables 3A,B), apart from mice lacking KCNJ15. The vacuolar area appeared to be marginally increased in the knockout mice (**Figure 8**, *p* < 0.01). The vacuolar pH for KCNJ15^-/-^ neutrophils was 0.1 pH unit higher than WT controls (*p* < 0.05).

#### Respiratory Burst Activity in Knockout Mouse Neutrophils

We confined our investigations of oxidase activity to mouse models that had either demonstrated a difference in vacuolar pH or area or had previously been cited to have an abnormality in the respiratory burst. The respiratory burst had previously been described as abnormally low in ClC3^-/-^ and KCC3^-/-^ neutrophils (Moreland et al., 2006; Sun et al., 2012) and in CLIC1^-/-^ macrophages (Jiang et al., 2012).

**FIGURE 9:**
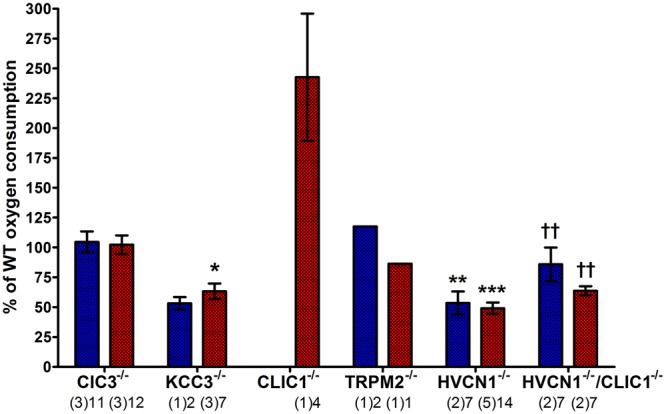
**Measuring oxygen consumption in ClC3, KCC3, CLIC1, TRPM2, and HVCN1 deficient mouse peritoneal neutrophils induced by PMA (blue) and opsonized *Candida* (red).** Oxygen consumption (OC) is presented as a relative percentage of the wild-type control (mean ± SEM). The numbers below indicate the number of separate experiments in brackets and number of wells recorded. Differences from WT neutrophils within each experiment: ^∗^*p* < 0.05, ^∗∗^*p* < 0.01, ^∗∗∗^*p* < 0.001. The statistics for HVCN1^-/-^/CLIC1^-/-^ neutrophils are compared to HVCN1^-/-^ controls where ††*p* < 0.01.

In the ClC3^-/-^ neutrophils, respiration had been found to be particularly compromised when they were stimulated with opsonised zymosan, a phagocytic stimulus, rather than the soluble agonist PMA (Moreland et al., 2006) although total oxidase response was also reduced with this agent. We were unable to reproduce this effect - we found that oxygen consumption induced by both PMA and the phagocytosis of heat-killed *Candida* in the knockout neutrophils was no different from control neutrophils (**Figure 9**).

We confirmed that the KCC3^-/-^ mouse neutrophils did have abnormal oxidase activity (Sun et al., 2012). The oxygen consumed is reduced to 53%, and 63% of normal after stimulation with PMA or opsonized *Candida* respectively. Oxidase activity in HVCN1^-/-^ neutrophils was again about half of normal (Levine et al., 2015).

The respiratory burst of neutrophils from both CLIC1^-/-^ and HVCN1^-/-^/CLIC1^-/-^ mice was greater than normal: for CLIC1^-/-^ approximately 242% (±53%) of WT control, and 86% (±14%) and 64% (±4%) for PMA and *Candida* stimulate respiration by HVCN1^-/-^/CLIC1^-/-^ neutrophils. These measurements were only made once on the CLIC1^-/-^ and twice on the HVCN1^-/-^/CLIC1^-/-^ mice. Although four wells were measured with each of the stimuli in both the control and knock-out mice the results must be interpreted with this caveat. The respiratory burst certainly did not appear to be depressed as it was in CLIC1^-/-^ macrophages (Jiang et al., 2012).

While we had found no differences in vacuolar pH and area between WT and TRPM2^-/-^ neutrophils, we wanted to assess the NOX2 activity as it has been reported that TRPM2 deficient neutrophils and macrophages have an increased production of reactive oxygen species (Di et al., 2012). We measured the PMA-induced oxygen consumption was 117% of the WT control, and *Candida*-induced oxygen consumption was 86% of WT control, which do not resemble the 2- to 3-fold increase reported by Di and co-workers in both conditions.

### Vacuolar pH and Area in Neutrophils of Patients with Channelopathies Appear Normal in CF Patients

**FIGURE 10:**
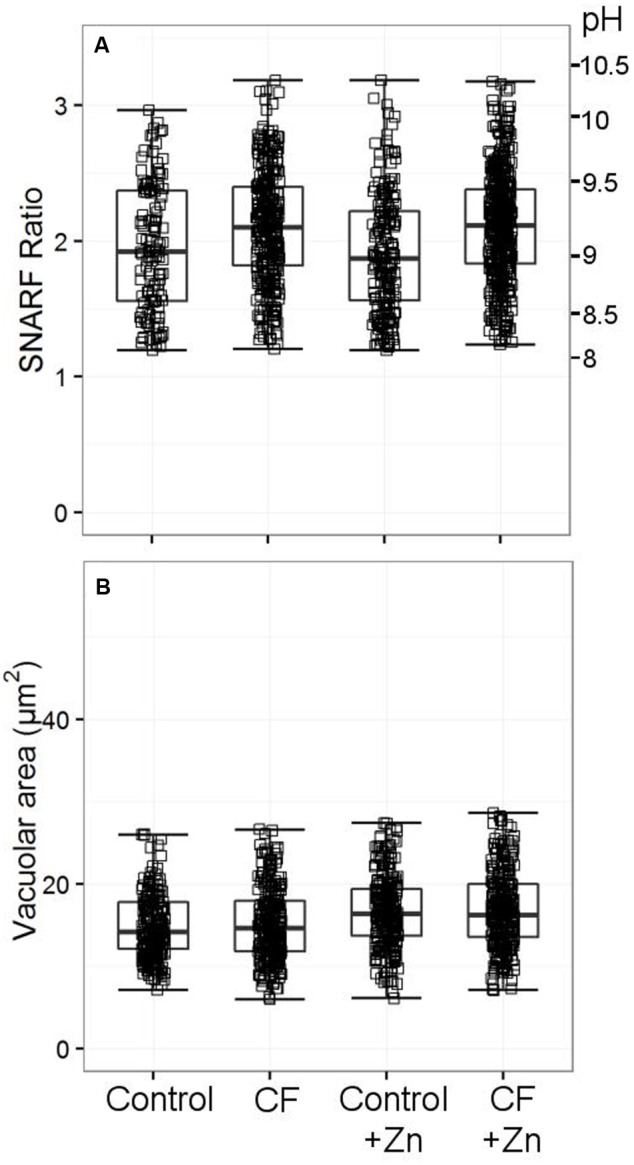
**Vacuolar pH (A)** and area **(B)** from two patients with cystic fibrosis (CF) with and without 300 μM zinc. Both patients were tested only once. Acidic vacuoles with a SFR value less than 1.2 were excluded. Between 120 and 440 vacuoles were counted for vacuolar pH, between 238 and 304 for the vacuolar area. No differences were observed between the healthy controls and the patients’ vacuolar parameters.

While it has been noted previously that patients with CF have abnormal neutrophil oxidase activity (Brockbank et al., 2005), we could find no abnormality in the vacuolar pH and area (**Figure 10**). We also measured cytoplasmic pH in reference to one claim that found abnormally high cytoplasmic pH in CF patients (Coakley et al., 2000) – again, we did not replicate this finding (Supplementary Table S4).

The vacuolar pH and area were also measured twice in two patients with mutations in the Bestrophin1 gene: patient 1 had total allelic deletion, while patient 2 had a deletion and a missense mutation in the other allele. Patient 1 had no detectable abnormalities in the vacuolar parameters, while for patient 2, the vacuolar area was larger with and without Zn^2+^. Two patients were tested with Albers-Schönberg disease or autosomal dominant osteopetrosis, caused by a mutation in ClC7. Patient 1 had lower vacuolar pH only with Zn^2+^, while patient 2 with Zn^2+^ had reduced vacuolar area, but the results on the whole were not grossly abnormal. Only one patient was tested once with the disorder in the MCOLN1 gene, or Mucolipidosis type IV, and had no significant changes in either parameter (Supplementary Table S5).

## Discussion

In order to identify the ion channels contributing to charge compensation in neutrophils, we have investigated the effects of known channel inhibitors and examined knockout mice and patients with non-functioning channels.

There has been considerable interest in the role of chloride channels in neutrophil function because of the belief that myeloperoxidase generates HOCl in this compartment, although this mechanism has been contested (Levine and Segal, 2016). The generation of HOCl would require the movement of Cl^-^ into the vacuole, which would be against the electrochemical gradient generated by the passage of electrons into the vacuole through NADPH oxidase. Neutrophils have a particularly high resting cytosolic concentration of Cl^-^; approximately 80 meq/L or 80 mM (Simchowitz and De Weer, 1986). After stimulation by soluble agonists (Yasuaki et al., 1993) or phagocytosis (Busetto et al., 2007), they release large quantities of Cl^-^ into the extracellular medium (Menegazzi et al., 1999), which is thought to adjust for phagocytosis-induced cellular swelling (Stoddard et al., 1993).

The classical broad spectrum Cl^-^ channel blockers, DCPIB and FFA both produced vacuolar acidification without inhibiting oxidase activity implying that they were preventing the efflux of Cl^-^ from the vacuole – it is known that DPI, an inhibitor of the oxidase, causes vacuolar acidification (Jankowski et al., 2002). FFA caused a slight increase in vacuolar volume, suggesting that it might be trapping Cl^-^ in the vacuole, although this was not the case for DCPIB. In the presence of Zn^2+^, which blocks proton channels, both these inhibitors induced vacuolar swelling more so than zinc alone and despite diminished oxidase activity, suggesting that the additional swelling resulted from the influx of non-proton cations, K^+^ and/or Na^+^. The effects observed with chloride channel inhibitors suggest that it is more likely that Cl^-^ will be passing out of the vacuole to compensate the charge than into it as the substrate for the generation of HOCl.

However, although primarily thought of as blockers of Cl^-^ channels, DCPIB and FFA are rather non-specific – as demonstrated in **Table 1**. DCPIB was first described as a “potent and selective” inhibitor of the swell-activated chloride current ICI_swell_ (Decher et al., 2001), or of the volume-regulated anion channel (VRAC), which is thought to participate in regulatory volume decrease (RVD) under certain physiological conditions. However, other DCPIB-sensitive ion channels have been reported. Sites of inhibition include inwardly rectifying K^+^ channels (Deng et al., 2016), and the K^+^/Cl^-^ co-transporter KCC3 (Adragna et al., 2015), while it has been shown to activate the 2P K^+^ channels TREK-1 and TREK-2 (Minieri et al., 2013). There is evidence that it has no effect on CFTR (Decher et al., 2001). FFA blocks ICI_swell_ (Jin et al., 2003), CFTR (McCarty et al., 1993) (but at a higher concentration than used in these studies), CLIC1 (Liantonio et al., 2007) and calcium-activated Cl^-^ channels (Oh et al., 2008), and some TRP channels (Guinamard et al., 2013). Both FFA and DCPIB open 2P K^+^ channels TWIK-1 and TWIK-2 (Takahira et al., 2005).

We thus attempted to identify a specific Cl^-^ channel by examining the function of neutrophils from humans and mice in which such channels were defective.

Two channels, in particular, have been proposed as conducting Cl^-^ into the vacuole; CFTR (Painter et al., 2010) and ClC3 (Nunes et al., 2013; Wang and Nauseef, 2015). Painter et al. (2010) described that the killing of *Pseudomonas aeruginosa* by neutrophils was impaired in cells from patients with CF and by normal neutrophils treated with GlyH-101, which they took to be a specific inhibitor of CFTR. They found bacterial killing to be marginally reduced by the CF patient’s cells and after treatment with 50 μM GlyH-101 (Painter et al., 2008). However, the experiment was conducted in Cl^-^ free extracellular medium for the first 10 min, and the effect of such treatment on CF cells was not established. In addition, Melis et al. (2014) found that GlyH-101 used at 50 μM reduced cell viability by over 50%. They also found that GlyH-101 almost completely blocked other Cl^-^ conductances including the volume-sensitive outwardly rectifying Cl^-^ conductance (VSORC) and Ca^2+^-dependent Cl^-^ conductance when used at 10 μM. We found no abnormalities neutrophils from CF patients with the common ΔF508 mutation, which argues against an essential role for this channel in charge compensation of the oxidase. Melis et al. (2014) also demonstrated that the pharmacological inhibitor, CFTR inh-172, is not specific so the small downward shift in vacuolar pH of human and HVCN1^-/-^ mouse neutrophils (Supplementary Table S5) produced by this agent is likely to be due to an off-target effect in the light of the normal results obtained with CF patient cells. We found no evidence of significant levels of expression of CFTR in the archival neutrophil mRNA expression data, but there is evidence for its expression in neutrophils, albeit at very low levels (Painter et al., 2006; McKeon et al., 2010).

Moreland et al. (2006) observed that neutrophils from ClC3 knock-out mice displayed markedly reduced NADPH oxidase activity in response to opsonized zymosan and modestly reduced activity after phorbol 12-myristate 13-acetate and thus inferred that ClC3 participates in charge compensation, although they did not provide a clear mechanism by which this might be achieved. We found oxidase activity in ClC3 knock-out mice to be entirely normal after stimulation with opsonized *Candida* or with PMA One difference between our experiments and those of Moreland et al. (2006) was that they used a chemiluminescence assay to measure superoxide production, whereas we measured oxygen consumption directly. Of perhaps more consequence is that we used ClC3^-/-^ mice generated by a different method to Moreland et al. (2006): our mice were generated by Jentsch and colleagues (Stobrawa et al., 2001) in which exon 3 was deleted, causing complete ablation of the gene in the resulting mice when tested with an antibody targeted to the N-terminus of the ClC3 gene made in-house. In contrast, part of exon 6 and all of exon 7 was removed in the other knockout mouse model, and the absence of the gene protein was tested using a commercial antibody targeted at the C-terminus (Dickerson et al., 2002) that Stobrawa et al. (2001) advised against using.

We found no impairment of vacuolar pH in CLIC1^-/-^ or HVCN1^-/-^/CLIC1^-/-^ bone marrow-derived neutrophils, as it has been noted in CLIC1^-/-^ peritoneal macrophages (Jiang et al., 2012) and bone marrow-derived dendritic cells (Salao et al., 2016). Most of the charge compensation of the neutrophil oxidase is conducted through the HVCN1 channel. We made the double KO mice because, although the HVCN1 proton channel is blocked by 100 μM Zn^2+^, this effect is only partial as evidenced by the relatively poorly elevated pH and vacuolar volume in Zn^2+^ treated cells as compared with HVCN1^-/-^ cells.

Instead, we observed a slight but significant decrease in vacuolar area in the double knockout HVCN1^-/-^/CLIC1^-/-^ mice compared to the HVCN1^-/-^ controls. We also tested oxidase activity in the double knockout mouse; oxygen consumption was significantly raised in the double knockout compared to HVCN1^-/-^ controls. If CLIC1 helps to compensate V-ATPase activity by matching proton influx with chloride influx on granule and phagosomal membranes, then its deletion might reduce water influx, and consequently vacuolar area, as was observed. Further investigation is necessary to confirm these results.

ICI_swell_, or the VRAC (Jentsch et al., 2016), is blocked by three of the four Cl^-^ channel inhibitors we found to reduce vacuolar pH (**Table 2**). Cells from a mouse in which LRRC8A was non-functional (Behe et al) had no effect on neutrophil vacuolar pH or volume, or on the respiratory burst.

There is also evidence that the entry of K^+^ into the phagocytic vacuole is driven by activity of the oxidase (Reeves et al., 2002) and the enhanced swelling of the vacuole induced by DCPIB and FFA described above could reflect the augmented entry of this ion induced by these agents when Cl^-^ efflux is replaced by K^+^ influx. We thus examined the effect of K^+^ channel blockers. The broad spectrum blockers, anandamide and quinidine, did change vacuolar conditions. Anandamide caused a decrease in both vacuolar pH and area, without inhibiting oxidase activity, suggesting that it was blocking the influx of non-proton cations. Their effects were exaggerated by the presence of Zn^2+^ which led to a halving of oxygen consumption. In contrast, quinidine reduced vacuolar volume but not pH, suggesting that it is a more selective inhibitor of K^+^ influx. It also had an effect on respiration, reducing it by about 20%. Anandamide is slightly more selective than quinidine, inhibiting the two-pore domain (2P) K^+^ channels TASK-1 (KCNK3) (Maingret et al., 2001) and TASK-3 (KCNK9) (Andronic et al., 2013), and some voltage-dependent K^+^ channels (Poling et al., 1996; Moreno-Galindo et al., 2010). Of particular interest is the claim made by Lee et al. (2006) that NOX4 modulates TASK-1 activity in oxygen-sensing cells. Perhaps it is similarly modulated by NOX2 in neutrophils. Quinidine is a relatively non-specific blocker of both Na^+^ (Hondeghem and Matsubara, 1988) and K^+^ channels (calcium-activated (Yatani et al., 1993; Wrighton et al., 2015), indicates that changes induced by quinidine do not distinguish between K^+^ and Na^+^, but prior knowledge (Reeves et al., 2002) supports the likelihood that it is K^+^ that is passing into the vacuole. Quinidine also blocks voltage-gated (Tatsuta et al., 1994; Attali et al., 1997), and two-pore domain (Patel et al., 2000; Wulff and Zhorov, 2008; Andronic et al., 2013) K^+^ channels, as well as the swell activated chloride current ICI_swell_ (Voets et al., 1996).

We then tested a wide range of more specific K^+^ channel inhibitors to try to identify the K^+^ channel, but none of these induced significant alterations in vacuolar conditions.

Once again, we examined several knock-out mice lacking specific K^+^ channels which were selected according to expression levels in human neutrophils and included KCNQ1, KCNE3, KCNJ2, KCNJ15, and various TRP channel-deficient mice. Transient receptor potential (TRP) channels are unselective for cations, a few of which have been associated with neutrophil function, including TRPC1 (Lindemann et al., 2015) and TRPM2 (Heiner et al., 2003a). TRPM2 has been described as a negative feedback mechanism to prevent excess ROS produced by the NADPH oxidase in phagocytes (Di et al., 2012) by regulating vacuolar membrane depolarisation through Ca^2+^ and Na^+^ ionic movement. We could not reproduce the enhanced respiratory burst reported in TRPM2^-/-^ mouse neutrophils (Di et al., 2012) and we found no changes in the vacuolar pH and area compared to controls. TRP channel blockers, such as amiodarone and SKF96365 (Tanahashi et al., 2016), were tested but were also without effect.

KCC3^-/-^ mice have been shown to have an impairment of NADPH oxidase activity with diminished bacterial killing ability (Sun et al., 2012), which we confirmed using the Seahorse assay instead of the lucigenin-enhanced chemiluminescence assays they employed, but were unable to detect alterations in vacuolar pH or area.

In summary, the broad spectrum channel blockers, DCPIB and FFA, anandamide and quinidine, affect vacuolar physiology by altering pH and cross-sectional area in different ways. DCPIB and FFA are largely considered to block Cl^-^ channels and their effects were consistent with charge compensation by the efflux of this ion from the vacuole. In contrast, anandamide and quinidine are largely considered to be blockers of K^+^ channels and their effects were consistent with the oxidase-induced flux of K^+^ into the vacuole. Even so, these inhibitors are not highly ion specific and the results must be interpreted with this in mind. Our inability to identify individual channels in these processes could result from our selection of the wrong selective inhibitors or knock-out animals or there might be considerable redundancy in the system, and for example the loss of a channel might result in exaggerated membrane depolarization to a level that would then open another voltage-gated channel that would not normally participate in physiological charge compensation. However, the effects of the four stated ion channel blockers confirm that fluxes of ions other than protons are important for the regulation of vacuolar pH and area neutrophil phagosomes.

## Author Contributions

Conception/design of work: AS, PB, AL, JF. Data acquisition: JF, AL. Data analysis: MF, JF, AS, PB, AL. Data interpretation: AS, JF, MF, PB, AL. Paper revisions: JF, AS, PB, AL, MF. Final approval: JF, AS, PB, AL, MF. Agreement of accountability: JF, PB, AL, MF, AS.

## Conflict of Interest Statement

The authors declare that the research was conducted in the absence of any commercial or financial relationships that could be construed as a potential conflict of interest.
