# Corrigendum: An Exploration of Charge Compensating Ion Channels across the Phagocytic Vacuole of Neutrophils

**DOI:** 10.3389/fphar.2017.00728

**Published:** 2017-10-11

**Authors:** Juliet R. Foote, Philippe Behe, Mathew Frampton, Adam P. Levine, Anthony W. Segal

**Affiliations:** Division of Medicine, Centre for Molecular Medicine, University College London, London, United Kingdom

**Keywords:** neutrophil, ion channel, NADPH oxidase, phagocytosis, chloride, potassium

In the original article, there was a mistake in Figure 10A as published. The vacuolar pH measurements for the CF patient and control+Zn were incorrectly swapped. The corrected Figure 10 appears below.

**Figure 10:**
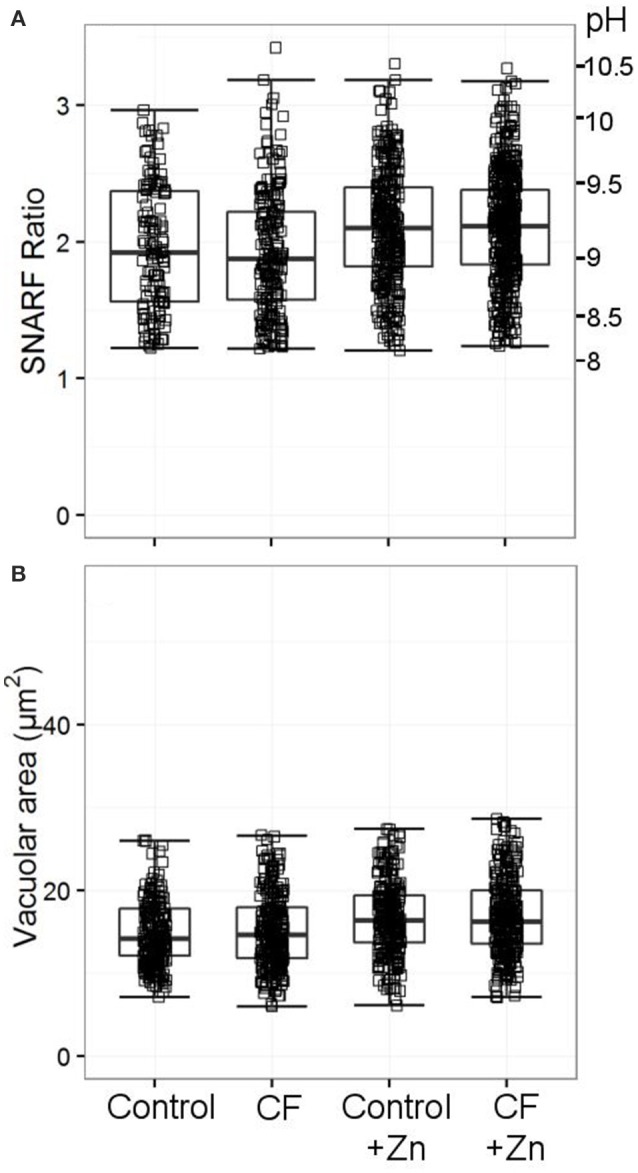
Vacuolar pH **(A)** and area **(B)** from two patients with cystic fibrosis (CF) with and without 300 μM zinc. Both patients were tested only once. Acidic vacuoles with a SFR value less than 1.2 were excluded. Between 120 and 440 vacuoles were counted for vacuolar pH, between 238 and 304 for the vacuolar area. No differences were observed between the healthy controls and the patients' vacuolar parameters.

In the original article, there was an error. We stated that there was no change in cytoplasmic pH in CF patients, but did not provide the data in the supplementary table.

A correction has been made to Results, Vacuolar pH and Area in Neutrophils of Patients with Channelopathies Appear Normal in CF Patients, paragraph 1:

While it has been noted previously that patients with CF have abnormal neutrophil oxidase activity (Brockbank et al., 2005), we could find no abnormality in the vacuolar pH and area (Figure 10). **We also measured the effect of the CFTR-inhibitor CFTR-172 on human, mouse WT and HVCN1**^−/−^ **neutrophils. The inhibitor caused a small decrease in vacuolar pH in human and HVCN1**^−/−^ **neutrophils (Supplementary Table 4)**.

Consequently, a correction has been made to Discussion, paragraph 6:

Two channels, in particular, have been proposed as conducting Cl^−^ into the vacuole; CFTR (Painter et al., 2010) and ClC3 (Nunes et al., 2013; Wang and Nauseef, 2015). Painter et al. (2010) described that the killing of Pseudomonas aeruginosa by neutrophils was impaired in cells from patients with CF and by normal neutrophils treated with GlyH-101, which they took to be a specific inhibitor of CFTR. They found bacterial killing to be marginally reduced by the CF patient's cells and after treatment with 50 μM GlyH-101 (Painter et al., 2008). However, the experiment was conducted in Cl^−^ free extracellular medium for the first 10 min, and the effect of such treatment on CF cells was not established. In addition, Melis et al. (2014) found that GlyH-101 used at 50 μM reduced cell viability by over 50%. They also found that GlyH-101 almost completely blocked other Cl^−^ conductances including the volume-sensitive outwardly rectifying Cl^−^conductance (VSORC) and Ca^2+^-dependent Cl^−^ conductance when used at 10 μM. We found no abnormalities in neutrophils from CF patients with the common ΔF508 mutation, which argues against an essential role for this channel in charge compensation of the oxidase. **However, we were only able to obtain samples from two patients, therefore these results must be confirmed in more patients to come to a significant conclusion**. Melis et al. (2014) also demonstrated that the pharmacological inhibitor, CFTR inh-172, is not specific so the small downward shift in vacuolar pH of human and HVCN1^−/−^ mouse neutrophils (Supplementary Table S4) produced by this agent is likely to be due to an off-target effect in the light of the normal results obtained with CF patient cells. We found no evidence of significant levels of expression of CFTR in the archival neutrophil mRNA expression data, but there is evidence for its expression in neutrophils, albeit at very low levels (Painter et al., 2006; McKeon et al., 2010).

In the published article, the citation for Supplementary Table S4 (BEST1, ClC7, MCOLN channelopathies data) should be replaced with Supplementary Table S5. Additionally, the citation for Supplementary Table S5 (CFTR-inh data) should be changed to Supplementary Table S4.

The authors apologize for these errors and state that this does not change the scientific conclusions of the article in any way.

## Conflict of interest statement

The authors declare that the research was conducted in the absence of any commercial or financial relationships that could be construed as a potential conflict of interest.
