# Supplementary material for: An Exploration of Charge Compensating Ion Channels across the Phagocytic Vacuole of Neutrophils
Source: Front Pharmacol. 2017 Feb 28;8:94. doi: 10.3389/fphar.2017.00094 (PMC5329019; doi:10.3389/fphar.2017.00094)
Supplement: Supplementary file 1 [file Table_1.DOCX]

**Table of tested inhibitors**

This table summarises the compound name, what concentration it was tested at, which channels it is stated to inhibit, if it were perceived to be toxic to the cells, and on-target and off-target effects.

| **Compound** | **Concentration** | **Inhibitor** | **Toxic** | **Effect on vacuolar pH and/or area** | **Off-target effects** |
| --- | --- | --- | --- | --- | --- |
| 293b-Chromanol | 20µM | K^+^ |  | None |  |
| 2APB | 20 - 100µM | TRP |  | Decrease in vacuolar pH and area | Inhibits NOX2 |
| 4-aminopyridine | 100µM - 1mM | K^+^ |  | None |  |
| 9-Phenanthrol | 20µM and 100µM | TRP |  | None |  |
| AC 5216 | 1nM - 3µM | TSPO |  | None |  |
| ACA | 10µM - 100µM | TRP |  | Decrease in vacuolar pH and area | Affects extracellular pH, inhibits degranulation |
| Acetozolamide | 1mM | K^+^ |  | None |  |
| Amiloride | 1mM | Na^+^ |  | Decrease in vacuolar pH | Affects extracellular pH |
| Amiodarone | 25µM and 50µM | K^+^ |  | None |  |
| Astemizole | 1µM | K^+^ |  | None |  |
| Bafilomycin | 1µM | V-ATPASE |  | None |  |
| BAPTA | 5µM | Ca^2+^ |  | None |  |
| Barium | 100µM | K^+^ |  | None |  |
| Benzamil | 10µM and 100µM | K^+^ | YES | Decrease in vacuolar pH |  |
| Bumetanide | 10µM and 100µM | K^+^ |  | None |  |
| Bupivacaine | 100µM - 500µM | K^+^ |  | None | Affects extracellular pH |
| CFTR-172 | 1µM - 10µM | Cl^-^ |  | Slight decrease in vacuolar pH |  |
| Chloroform | 800µM | Na^+^ |  | Decrease in vacuolar pH |  |
| Clofilium | 3µM - 300µM | K^+^ |  | None |  |
| Clotrizamole | 20µM | K^+^ |  | None |  |
| CyPPA | 30µM | K^+^ |  | None |  |
| Desipramine | 10µM and 100µM | K^+^ |  | None |  |
| DIDS | 1µM – 1mM | Cl^-^ | YES | None | Inhibits phagocytosis |
| DIOA | 5µM – 100µM | K^+^/Cl^-^ exchanger |  | None |  |
| DMA (dimethyl amiloride) | 300µM | Na^+^/H^+^ exchanger |  | Slight decrease in vacuolar pH |  |
| Dofetilide | 1µM | K^+^ |  | None |  |
| E4031 | 1µM | K^+^ |  | None |  |
| Erastin | 10µM and 50µM | TRP |  | Slight increase in vacuolar pH |  |
| Flecanide | 100µM | K^+^ |  | None |  |
| Furosemide | 1mM | K^+^ |  | None |  |
| Gadolinium | 10µM and 100µM | K^+^ |  | None |  |
| Halothane | 3% | K^+^ |  | None |  |
| HMR1556 | 1µM | K^+^ |  | None |  |
| IAA94 | 2µM - 50µM | Cl^-^ |  | None |  |
| KCN | 10µM – 1mM | Ca^2+^ |  | None |  |
| KR32568 | 5µM | TRP |  | None |  |
| Lidocaine | 300µM and 1mM | K^+^ |  | None |  |
| LOE908 hydrochloride | 1.25µM | K^+^ |  | Slight decrease in vacuolar pH |  |
| Lq2 (component of *Leiurus quinquestriatus* scorpion venom) | 20nM | K^+^ |  | None |  |
| Maurotoxin | 50-400nM | K^+^ |  | None | Decreased phagocytosis |
| Mexiletine | 10µM and 100µM | K^+^ |  | None |  |
| ML204 | 20µM and 100µM | K^+^ |  | None |  |
| NPPB | 30µM - 300µM | Cl^-^ |  | Decreases vacuolar pH | Acts as a protonophore |
| NS8593 | 1µM – 100µM | K^+^ |  | None |  |
| Ouabain | 10µM | K^+^ |  | None |  |
| PD118057 | 10µM | K^+^ | YES | Decreases vacuolar pH | Decreased phagocytosis |
| Phloretin | 10µM - 300µM | Cl^-^ |  | Decreases vacuolar pH | Inhibits degranulation, affects external pH |
| PK-THPP | 3µM - 30µM | K^+^ |  | None |  |
| Procainamide | 50µM | K^+^ |  | None |  |
| Psora-4 | 10µM - 100µM | K^+^ |  | None |  |
| Quinine | 30µM - 300µM | K^+^ |  | Slight decrease in vacuolar pH and area |  |
| Ranolazine | 50µM | K^+^ |  | None |  |
| Ruthenium red | 1µM and 10µM | Ca^2+^, Na^+^ |  | None |  |
| SITS | 30nM - 10µM | Cl^-^ | YES | None |  |
| SKF96365 | 20µM - 100µM | K^+^ |  | None |  |
| Spadin | 1µM and 3µM | K^+^ |  | None |  |
| Stichodactyla | 5nM | K^+^ |  | None |  |
| Tamoxifen | 3µM - 100µM | Cl^-^ | YES | Slight decrease in vacuolar pH | Inhibits phagocytosis |
| Talniflumate | 100µM and 200µM | Cl^-^  and Cl^-^/HCO3^-^ exchanger |  | None |  |
| TEA | 10mM | K^+^, Na^+^ |  | None |  |
| Terfenadine | 1µM | K^+^, Ca^2+^ |  | None |  |
| Tetrodotoxin | 1µM and 5µM | Na^+^ |  | None |  |
| Thioridazine | 1µM and 50µM | K^+^ |  | None |  |
| TRAM-34 | 1µM - 300µM | K^+^ |  | None |  |
| TRO19622 | 50µM and 100µM | TRP |  | None |  |
| UK5099 | 62.5µM – 1mM | Na^+^ |  | None |  |
| WW781 | 10µM | Cl^-^ |  | None |  |
