# Supplementary material for: An Exploration of Charge Compensating Ion Channels across the Phagocytic Vacuole of Neutrophils
Source: Front Pharmacol. 2017 Feb 28;8:94. doi: 10.3389/fphar.2017.00094 (PMC5329019; doi:10.3389/fphar.2017.00094)
Supplement: Supplementary file 2 [file Table_2.DOCX]

Studies of human neutrophil gene expression

The details for each individual study sampled in the composite neutrophil expression data are recorded below.

| Title of study | Source link | Number of samples per study |
| --- | --- | --- |
| Transcription profiling of human neutrophil and PBMC gene expression data from Jobs Syndrome individuals | <https://www.ebi.ac.uk/arrayexpress/experiments/E-GEOD-8507> | 11 |
| Transcription profiling by array of human neutrophils after 30 minutes' exercise | <https://www.ebi.ac.uk/arrayexpress/experiments/E-GEOD-8668> | 24 |
| mRNA expression profiling of human immune cell subsets (HUG) | <https://www.ebi.ac.uk/arrayexpress/experiments/E-GEOD-28491> | 5 |
| Transcription profiling by array of human peripheral blood mononuclear cells after treatment with community-associated Staphylococcus aureus and incubation for different lengths of time | <https://www.ebi.ac.uk/arrayexpress/experiments/E-GEOD-16837> | 4 |
| Transcription profiling by array of human bone marrow CD34+ cells, promyelocytes and neutrophils, as well as PR-9 and NB-4 cell lines, to investigate acute myeloid leukemia | <https://www.ebi.ac.uk/arrayexpress/experiments/E-GEOD-12662> | 5 |
| mRNA expression profiling of human immune cell subset (Roche) | <https://www.ebi.ac.uk/arrayexpress/experiments/E-GEOD-28490> | 3 |
| Transcription profiling by array of human neutrophils isolated via microfluidics after treatment with either lipopolysaccharide or granulocyte-macrophage colony-stimulating factor and interferon gamma | <https://www.ebi.ac.uk/arrayexpress/experiments/E-GEOD-22103> | 4 |
| Expression profiles from a variety of resting and activated human immune cells | <https://www.ebi.ac.uk/arrayexpress/experiments/E-GEOD-22886> | 5 |
| Transcription profiling of human neutrophils obtained after exposure endotoxin by bronchoscopic instillation reveals differential gene expression between air space and circulating neutrophils | <https://www.ebi.ac.uk/arrayexpress/experiments/E-GEOD-2322> | 5 |
