# Supplementary material for: An Exploration of Charge Compensating Ion Channels across the Phagocytic Vacuole of Neutrophils
Source: Front Pharmacol. 2017 Feb 28;8:94. doi: 10.3389/fphar.2017.00094 (PMC5329019; doi:10.3389/fphar.2017.00094)
Supplement: Supplementary file 3 [file Table_3.DOCX]

The following tables present results from testing differences between WT and knockout mouse models. N = number of experiments performed for each condition, while no. of points = number of individual experiments.

**Knockout mice**

1. **Vacuolar pH**

| **N** | **Control** | **No. of points** | **Control median (SFR/pH)** | | **Knockout** | **No. of points** | **Knockout median (SFR/pH)** | | **p value** |
| --- | --- | --- | --- | --- | --- | --- | --- | --- | --- |
| 1 | WT | 394 | 0.88 | 7.4 | ClC4 | 350 | 0.90 | 7.5 | 0.060 |
| 1 | WT | 512 | 1.07 | 7.9 | ClC7 | 183 | 1.04 | 7.8 | 0.029 |
| 3 | WT | 371 | 1.04 | 7.8 | KCNQ1 | 311 | 1.13 | 8.0 | 0.013 |
| 1 | WT | 98 | 0.92 | 7.5 | KCNE3 | 206 | 0.72 | 6.7 | 5.0E-5 |
| 1 | WT | 410 | 1.00 | 7.7 | TRPA1/V1 | 412 | 1.00 | 7.7 | 4.0E-4 |
| 1 | WT | 399 | 0.86 | 7.3 | TRPV2 | 298 | 0.89 | 7.4 | 1.0E-6 |
| 1 | HVCN1 | 141 | 1.88 | 9.0 | HVCN1/KCNQ1 | 63 | 1.63 | 8.7 | 2.0E-5 |

1. **Vacuolar area**

| **N** | **Control** | **No. of points** | **Control median** | **Knockout** | **No. of points** | **Knockout median** | **p value** |
| --- | --- | --- | --- | --- | --- | --- | --- |
| 1 | WT | 186 | 10.71 | ClC4 | 200 | 10.40 | 0.064 |
| 1 | WT | 112 | 13.23 | ClC7 | 30 | 13.71 | 0.421 |
| 3 | WT | 162 | 11.66 | KCNQ1 | 189 | 11.34 | 0.943 |
| 1 | WT | 34 | 10.16 | KCNE3 | 79 | 10.56 | 0.117 |
| 1 | WT | 79 | 11.66 | TRPA1/V1 | 119 | 12.29 | 0.48 |
| 1 | WT | 174 | 12.37 | TRPV2 | 178 | 12.92 | 0.047 |
| 1 | HVCN1 | 78 | 17.09 | HVCN1/KCNQ1 | 16 | 16.31 | 0.648 |
