# Supplementary material for: An Exploration of Charge Compensating Ion Channels across the Phagocytic Vacuole of Neutrophils
Source: Front Pharmacol. 2017 Feb 28;8:94. doi: 10.3389/fphar.2017.00094 (PMC5329019; doi:10.3389/fphar.2017.00094)
Supplement: Supplementary file 4 [file Table_4.DOCX]

The following tables present results from testing differences between healthy controls and patients with certain channelopathies. N = number of experiments performed for each condition, while no. of points = number of individual experiments.

**Patients with channelopathies**

1. **Vacuolar pH**

| N | Control | No. of points | Control median (SFR/pH) | | Patient | No. of points | Patient median (SFR/pH) | | p value |
| --- | --- | --- | --- | --- | --- | --- | --- | --- | --- |
| 2 | HC | 444 | 1.8 | 8.9 | BEST1 (P1) | 503 | 1.8 | 8.9 | 0.663 |
| 2 | HC+Zn | 340 | 2.0 | 9.0 | BEST1 (P1)+Zn | 281 | 2.0 | 9.0 | 0.581 |
| 2 | HC | 747 | 1.7 | 8.8 | BEST1 (P2) | 729 | 1.7 | 8.8 | 0.042 |
| 2 | HC+Zn | 433 | 1.8 | 8.9 | BEST1 (P2)+Zn | 549 | 1.7 | 8.8 | 0.432 |
| 1 | HC | 401 | 1.2 | 8.1 | ClC7 (P1) | 401 | 1.2 | 8.1 | 0.170 |
| 1 | HC+Zn | 642 | 1.7 | 8.8 | ClC7 (P1)+Zn | 641 | 1.3 | 8.3 | 0.002 |
| 1 | HC | 201 | 1.2 | 8.1 | ClC7 (P2) | 202 | 1.2 | 8.1 | 0.096 |
| 1 | HC+Zn | 204 | 2.0 | 9.1 | ClC7 (P2)+Zn | 202 | 2.0 | 9.1 | 0.360 |
| 1 | HC | 282 | 1.9 | 9.0 | MCOLN | 415 | 1.9 | 9.0 | 0.391 |

1. **Vacuolar area**

| N | Control | No. of points | Control median | Patient | No. of points | Patient median | p value |
| --- | --- | --- | --- | --- | --- | --- | --- |
| 2 | HC | 471 | 14.50 | BEST1 (P1) | 381 | 14.50 | 0.833 |
| 2 | HC+Zn | 258 | 15.83 | BEST1 (P1) +Zn | 256 | 15.44 | 0.130 |
| 2 | HC | 612 | 15.68 | BEST1 (P2) | 660 | 16.86 | 0.003 |
| 2 | HC+Zn | 306 | 16.78 | BEST1 (P2) +Zn | 355 | 17.8 | 0.006 |
| 1 | HC | 400 | 14.73 | ClC7 (P1) | 400 | 14.18 | 0.003 |
| 1 | HC+Zn | 404 | 17.8 | ClC7 (P1) +Zn | 401 | 18.43 | 0.390 |
| 1 | HC | 200 | 11.97 | ClC7 (P2) | 197 | 12.76 | 0.066 |
| 1 | HC+Zn | 201 | 15.76 | ClC7 (P2) +Zn | 200 | 14.73 | 0.017 |
| 1 | HC | 214 | 12.92 | MCOLN | 245 | 13.39 | 0.072 |
