# Supplementary material for: Corrigendum: An Exploration of Charge Compensating Ion Channels across the Phagocytic Vacuole of Neutrophils
Source: Front Pharmacol. 2017 Oct 11;8:728. doi: 10.3389/fphar.2017.00728 (PMC5641588; doi:10.3389/fphar.2017.00728)
Supplement: Supplementary file 1 [file Table4.DOCX]

**Effect of CFTR-172 inhibitor on vacuolar pH**

| N | Control | No. of points | Control median | | Inhibitor concentration | No. of points | Inhibitor median | | p value |
| --- | --- | --- | --- | --- | --- | --- | --- | --- | --- |
| 1 | HC | 105 | 2.1 | 9.2 | 5uM | 92 | 1.6 | 8.7 | 9.20E-05 |
| 2 | Wt | 170 | 0.9 | 7.4 | 5uM | 127 | 0.9 | 7.4 | 0.368 |
| 4 | HVCN1 | 589 | 2.4 | 9.5 | 5uM | 617 | 2.2 | 9.3 | 1.50E-23 |
